# One hotspot *RB1* mutation disrupt *RB1* function founded in a Chinese patient

**DOI:** 10.3389/fonc.2026.1728083

**Published:** 2026-05-26

**Authors:** Yan Liu, Kexin Ren, Fanglin He, Yuan Deng, Yixiong Zhou, Xiaolin Huang, Leilei Zhang

**Affiliations:** 1 Department of Ophthalmology, Ninth People’s Hospital, Shanghai JiaoTong University School of Medicine, Shanghai, China; 2 Shanghai Key Laboratory of Orbital Diseases and Ocular Oncology, Shanghai, China

**Keywords:** hotspot, mutation, p.E125*, Rb1, retinoblastoma

## Abstract

**Introduction:**

Retinoblastoma is divided into two clinical types, sporadic and heritable, and is the most common primary intraocular malignancy in infancy and childhood. It was the first malignancy to demonstrate the genetic etiology of cancer, with the *RB1* gene as the only pathological gene present in heritable cases. Although the *RB1* mutation p.E125* had been previously reported in other retinoblastoma patients, it lacked functional analysis.

**Methods:**

In this study, we identified the *RB1* p.E125* mutation in a bilateral retinoblastoma patient from China. We investigated the distribution, cell localization, and function of this mutation using molecular biology and structural analysis following the transfection of cells with plasmids encoding the mutant *RB1* gene.

**Results:**

Functional analyses revealed abnormal protein localization, altered cell cycle distribution, and apoptosis in cells transfected with the mutant *RB1* plasmids.

**Discussion:**

Our findings contribute to a better understanding of *RB1* mutation hotspots. Furthermore, our results highlight the importance of offering targeted genetic testing and counseling to families with *RB1* mutations. The identification of the somatic origin of this mutation was vital in ruling out the heritability of this condition in this specific patient.

## 1 Introduction

Retinoblastoma [MIM 180200; RB] (1) is the most common intraocular malignancy in children and represents 3% of all childhood malignancies (2), with an incidence ranging from 1 in 16,000 to 1 in 18,000 live births; approximately 8,000 new cases are predicted each year (3). There is no validated geographic predisposition or racial variations for the development of RB. RB is currently split into two clinical types: sporadic and heritable. All bilateral RB cases are heritable, but only a small proportion of unilateral cases can be passed on to future generations (4). Mean age at diagnosis is approximately 27 months for unilateral retinoblastoma and 15 months for bilateral cases (5), although there are some differences in different regions. If RB is confined to the eye and noticed early, prompt treatment could cure the cancer and save the eye (or eyes) and vision. However, delayed diagnosis could lead to metastasis elsewhere in the body. Although RB that metastasized out of the eye could be cured by extensive intervention; complete curing is rare (6).

The *RB1* gene is located in region 14 of chromosome 13 (7). It was the first tumor suppressor gene to be identified that contained 27 exons, distributed over 200 kb of DNA, encoding a 4.7 kb mRNA that translates into a 928-amino-acid-long protein [RB protein; NP_000312] (8). Nonsense mutations constitute a significant portion of *RB1* pathogenic variants. These point mutations result in a premature stop codon, generally leading to mRNA degradation via nonsense-mediated decay or the production of a truncated, non-functional protein lacking critical domains for tumor suppression. Approximately 60% of *RB1* nonintermediary germ line mutations could result in sporadic cases of unilateral disease, with no family history of RB. These *RB1* mutations arise locally within the developing retina (9). However, once an *RB1* mutation is present in the germline, it could result in the hereditary transmission of this disease. Individuals with hereditary RB have a 50% chance of passing these mutations on to their offspring. In addition, the risk of second malignant neoplasms was highest among RB patients who treated with radiotherapy (10). It had been reported that osteosarcoma was the most common second malignancy (11).

Knowledge of *RB1* gene mutations is important for genetic counseling and the characterization of phenotypic-genotypic relationships. To date, more than 900 *RB1* germline mutations have been published, and the recurrent *RB1* mutation is particularly important (12). In this study, we performed genetic screening of Chinese RB patients and their family members for heritable *RB1* mutation, and direct sequencing and phenotypic-genotypic relationships analysis were used to analyze the p.E125* mutation and explore the potential *RB1* mutation hotspot.

## 2 Materials and methods

### 2.1 Ethics statement

This study was approved by the Institutional Ethics Committee of Shanghai Ninth Hospital. Genetic counseling was provided to all participants. Informed written consent was obtained from the patient’s parents and each family member prior to participation in the study, in accordance with the Declaration of Helsinki.

### 2.2 Subjects and DNA extraction

All DNA samples were obtained from the Department of Shanghai Ninth Hospital. Genetic counseling was provided to the participants. According to the authority of the Institutional Ethics Committee of Shanghai Ninth Hospital, informed consent was obtained from each family member prior to participation in the study. Fresh venous blood samples were collected from the patients and their family members and 100 individuals with neither a personal nor family history of RB. The protocol of blood collection and genomic DNA extraction were conducted as described before (13).

### 2.3 PCR and sequencing

Direct polymerase chain reaction (PCR) sequencing of the 27 coding exons and their flanking intron regions was performed to screen for *RB1* mutations. The primer sets, PCR annealing temperatures and PCR reactions have been previously described (13). Family members were also screened.

### 2.4 Molecular modeling

To predict the possible impact of a substitution on the structure and function of the RB protein, a three-dimensional computer model was used to analyze the structural positioning of the mutation site. The crystal structure of the DNA-binding domain of RB protein was obtained from the Protein Data Bank (PDB) (code, 4ELJ, chain Å). As described, according to the basis of the SWISS-MODEL, the wild-type and mutant domain of the RB protein was modeled separately (13, 14).

### 2.5 Construction of the expression vectors

Wild-type *RB1* gene plasmid was obtained from Professor Zhao (Southern Medical University, Key Laboratory for Proteomics of in Guangdong Province). Then, the mutant and wild-type *RB1* with or without an EGFP tag were constructed. For EGFP-tagged plasmids, the EGFP sequence was inserted and fused specifically to the C-terminus of the *RB1* coding sequence. Plasmids without the EGFP tag were used for FITC/Annexin V apoptosis staining analysis of cell cycle distribution, while the plasmids with the EGFP tag were used for subcellular location analysis. The primers for the mutant p.E125* are (F1: GAAAAACATATAAATCAGTGTCCATAAATTCTTTAACTTACTAA; R1: ATGGACACTGATTTATATGTTTTTCTGTAGCTCAGTA);. The plasmid expression vectors were sequenced and confirmed to be without other mutations.

### 2.6 Cell culture and transfection

The Saos-2 and 293T cells were maintained in Dulbecco’s Modified Eagles Medium (DMEM; Gibco, CA, USA), supplemented with 10% fetal bovine serum (FBS; Gibco–Invitrogen, Grand Island, NY, USA) in a 5% CO2 atmosphere. A total of 2 × 105 cells were plated in 2 ml of growth medium and transfected after the cells were 60–80% confluent. Transfection was performed using Lipofectamine 2000 (Invitrogen, CA, USA), according to the manufacturer’s instructions.

### 2.7 Cell cycle and apoptosis analysis

Cell cycle assay and apoptosis assay were performed as described previously (13). Cell cycle distribution was detected and analyzed using the FACScan instrument and CellQuest program (Becton Dickinson, NJ, USA). Apoptosis samples were examined by the FACScan instrument (Becton Dickinson, NJ, USA), and data were analyzed using the CellQuest programs (Becton Dickinson, NJ, USA).

### 2.8 Detection of fluorescence

293T cells transfected with plasmids encoding wild-type *RB1* or the p.D125X *RB1* mutant were seeded onto glass coverslips (VWR, West Chester, PA, USA) in 12-well plates and cultured overnight. After transfection, cells were fixed in 4% (w/v) paraformaldehyde (Sigma-Aldrich, St. Louis, MO, USA) for 15 min at room temperature. The coverslips were washed again, and the nuclei were counterstained with 4´, 6-diamidino-2-phenylindole (DAPI; Vector Laboratories, Burlingame, CA, USA) for 2 min at room temperature. Fluorescent cells were visualized using a fluorescence microscope (Olympus BX51, Japan) for the plasmid containing the EGFP, which emit green fluorescence.

## 3 Results

### 3.1 Clinical characteristics and genetic analysis

**Figure 1:**
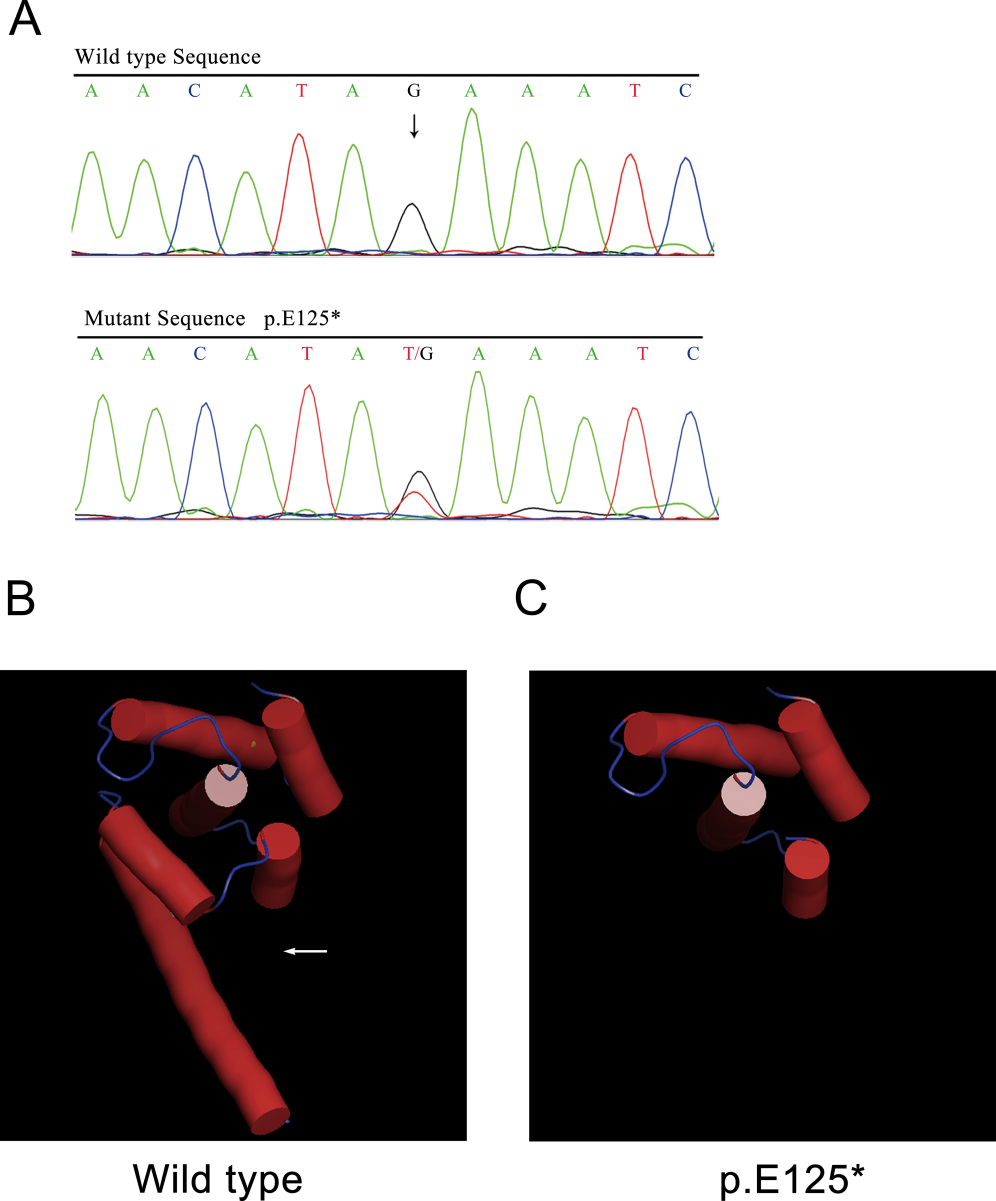
Wild-type and mutant plasmid sequence and molecular modeling of p.E125*. **(A)** part of the DNA sequence of the wild-type and mutant p.E125* are shown. **(B, C)** the molecular structures of the wild-type and p.E125*. The molecular modeling of p.E125* is shown in **(C)** and includes the amino acid substitution and premature termination, and the mutated regions are marked with white arrows indicating the missing molecular structure of the mutant *RB1* (p.E125*). The corresponding wild-type region around p.E125* is shown in **(B)**.

The proband was a 6-month-old Chinese male patient who presented clinically with bilateral leukocoria. Ophthalmic examination confirmed bilateral retinoblastoma. DNA from the RB patients were analyzed using reverse transcription PCR and the *RB1* gene was sequenced. We found a single base substitution, p.E125* (c.373G>T), in one Chinese RB patient, which led to nonsense amino acid changes with only 125 amino acids in the mutant, while the wild-type was 926 amino acids, which we previously described in detail (**Figure 1A**) (13). Peripheral blood DNA testing of the patient’s asymptomatic parents revealed no such mutation, indicating that this is a *de novo* germline mutation in the proband. The NCBI, dbSNP, and UCSC databases, along with the Leiden Open Variation Database (LOVD), confirm that codon 125 is a highly recurrent mutation site across multiple unrelated families globally. This is supported by independent reports from other research groups (15, 16), and our own previous identification of this exact variant in a separate Chinese cohort (13), establishing it as an *RB1* hotspot. In addition, a deletion at this site has been reported previously (17). Finally, it demonstrated that codon 125 is a recurrent mutation site in the *RB1* gene, which is a hotspot site. However, there was a non-associated functional analysis provided for the p.E125* mutation.

### 3.2 Molecular modeling of the pRB

For further analysis, a three-dimensional structural model was used to perform the computational analysis of this mutated protein. Compared with the wild-type *RB1*, the spatial structure of the mutated protein, p.E125*, was significantly shortened. The white arrow indicates the part of the wild-type protein that was deleted in the mutant (**Figures 1B, C**). This nonsense mutation causes the absence of the RB protein pocket structure and the following C-terminal part.

### 3.3 Subcellular location and loss of NLS

**Figure 2:**
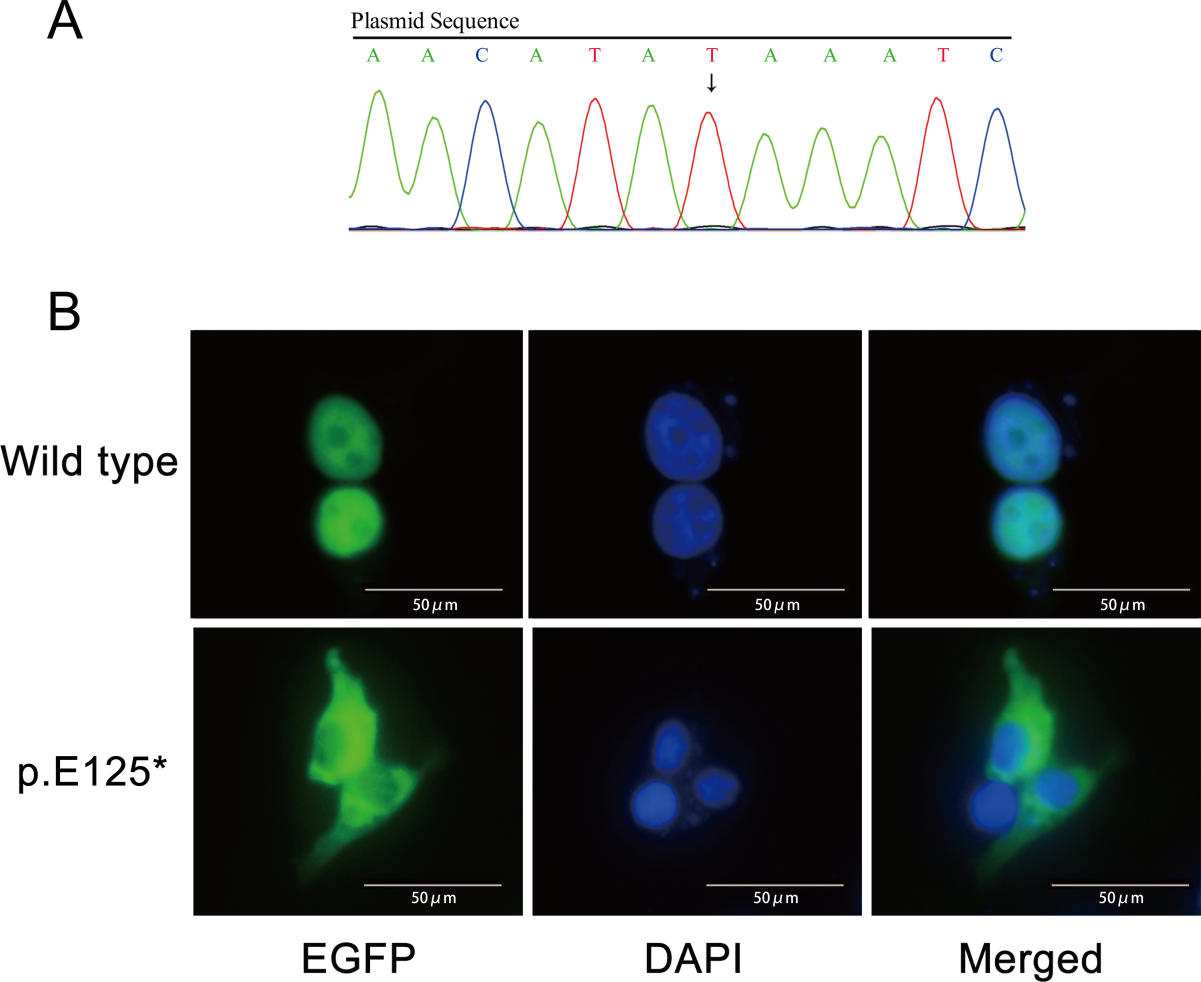
Expression and location of wild-type and mutant *RB1* in transfected Saos-2 cells. **(A)** the partial plasmid sequence of the mutant *RB1*, p.E125*. **(B)** the subcellular locations of the wild-type and mutant proteins determined by fluorescence after transfection in 293T cells. The left panel corresponds to the representative subcellular localization of RB as a fusion protein with an enhanced green fluorescent protein (EGFP) tag. The middle panel corresponds to nuclear staining with DAPI. The right panel is a merged image of the previous two images. Nuclear fluorescence was obtained with the wild-type protein RB1-EGFP. After transfection of RB-G125X-EGFP, cytoplasmic mislocalization and nuclear aggregation were observed in 293T cells but not in cells transfected with wild-type-RB-EGFP.

To explore the subcellular location of the mutant *RB1*, the mutant plasmid p.E125* was transfected into Saos-2 cells (**Figure 2A**). As shown in **Figure 2B**, wild-type RB protein was localized exclusively in the nucleus in a diffuse manner. In contrast, cells transfected with the mutant construct did not display a wild-type distribution, which is mainly distributed in the cytoplasm (**Figure 2B**). As we know, a gene exercises its function only when localized properly. Abnormal localization means it could not function normally. The nuclear localization of the normal RB protein relies on a bipartite nuclear localization signal (NLS) situated in the C-terminus (amino acids 860–876). Because the p.Glu125X mutation causes premature termination at amino acid 125, the resulting truncated protein lacks this critical NLS, rendering it unable to translocate to the nucleus where it normally functions.

### 3.4 Cell cycle distribution and apoptotic function of the mutants

**Figure 3:**
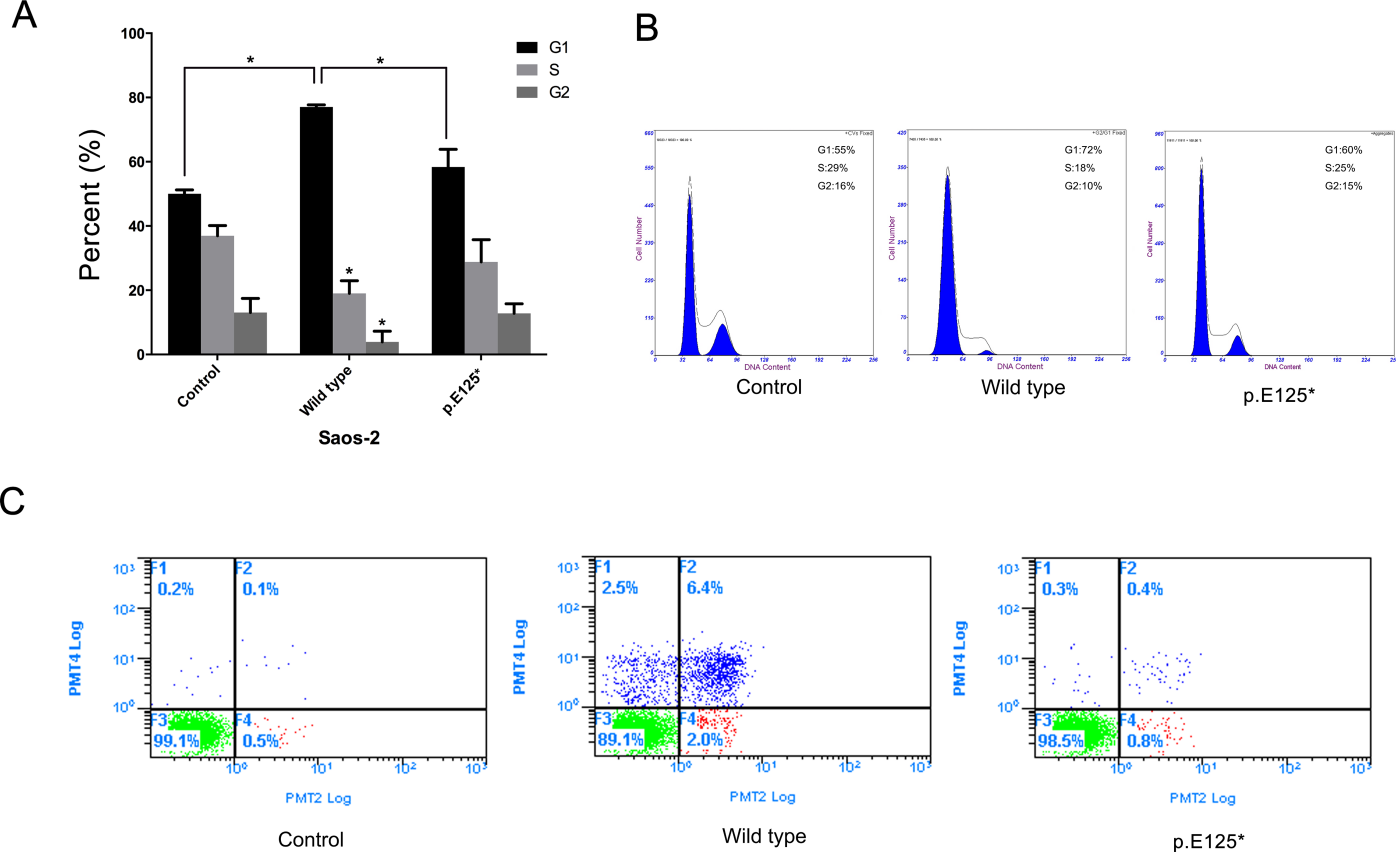
Differential effects of wild-type and mutant *RB1* on the cell cycle and apoptosis. **(A)** the cell cycle distribution was analyzed, and the histogram shows that transfection with the wild-type *RB1* plasmid significantly inhibited the cell progression, while transfection of the mutants did not. **(B)** cell cycle analysis indicated that the wild-type *RB1* promotes G1 phase arrest, whereas mutant *RB1* induces S phase. **(C)** the apoptosis rates of the 293T cells are shown in the negative control, wild-type *RB1* and p.E125* cells. After transfection with wild-type *RB1*, the proportion of late apoptosis increased from 0.6% (F2+F4) to 8.4% (F2+F4) in Saos-2 cells (p<0.05); however, these levels were nearly unchanged in cells transfected with p.E125* (1.2%) (*p<0.05). All experiments were repeated at least three times.

Because of the abnormal subcellular localization and the altered molecular model, the molecular function of p.E125* was further explored. Cell cycle distribution was examined through flow cytometry after transfection with the plasmids. After 48 h, approximately 72% of the cells transfected with the wild-type *RB1* plasmid were blocked in G1 phase, compared with the control cultures that showed 55% of cells in G1 phase. However, the percentage of cells in G1 phase was approximately 60% in the cells transfected with the mutant plasmid, p.E125* (p<0.05; **Figures 3A, B**). In addition, transfection with equal amounts of the p.E125* plasmid did not promote apoptosis compared with the control group, while the wild-type *RB1* could lead to an increase in apoptosis in cells. It suggested that the mutant *RB1* plasmid did not function as the wild-type *RB1*, which functions as a tumor suppressor.

## 4 Discussion

Mutations in codon 125 had been reported previously (13, 17). First, a deletion mutation was detected in codon 125 (17). Then, we found a nonsense mutation, p.E125* (c.373G>T) in codon 125, which had been previously observed in a Chinese family by our team (13). However, the related functional experiments were not conducted. This time, we performed the first thorough functional analysis of the mutant p.E125* (c.373G>T). It demonstrated that codon 125 may be a recurrent mutation site in the *RB1* gene, which plays an important role in the tumorigenesis of hereditary retinoblastoma.

The *RB1* gene codes for the retinoblastoma protein, which regulates cellular proliferation through binding to the E2F family of transcription factors, then represses genes related to cell proliferation or chromatin remodeling proteins. RB also reportedly upregulates p27 gene expression, which is implicated in cell differentiation, apoptosis, genomic integrity and chromosome stability (18–23). RB proteins contain several different domains. The A–B pocket domain is the major focus of tumorigenic mutations in the RB protein, which extends from amino acids 379 to 792. The pocket domain is required to arrest cell cycle progression through cell cycle-dependent phosphorylation and dephosphorylation (24). In this study, we found that the mutation of p.E125* led to the premature termination of the RB protein with 125 amino acids.

Because this truncation completely deletes the pocket domain, the mutant protein fundamentally loses its physical capacity to bind and inhibit E2F. Consequently, E2F is constitutively released, driving unregulated downstream target gene transcription and uncontrolled cell cycle progression from G1 into S phase. Additionally, we demonstrated that the p.Glu125X mutation abolishes the C-terminal bipartite nuclear localization signal (NLS, aa 858-868). Without this NLS, the truncated protein accumulates aberrantly in the cytoplasm (**Figure 2B**), further physically isolating it from any nuclear transcription factors it might otherwise interact with. Consequently, as evidenced by our flow cytometry data, the p.Glu125X mutant entirely fails to induce G1 phase arrest or promote apoptosis, confirming its complete loss of anti-oncogenic capability (**Figure 3**). While our Annexin V flow cytometry data clearly outlines the apoptotic deficit of this mutant, we acknowledge that future studies utilizing western blotting for Caspase 3 cleavage and pRb hypophosphorylation status would provide further nuanced insights into the ablated molecular pathways.

It has been reported that *RB1* gene deletion positively correlates with an advanced stage at diagnosis, a higher incidence of aggressive histopathological features and metastasis (25). In addition, the previous study found that leukocoria was the first clinical manifestation in RB patients with *RB1* mutations. However, RB patients without mutations manifested in many different forms, including weak eyes, decreased vision, and strabismus (13). Optical coherence tomography (OCT), as a non-contact and non-invasive technique, can be used to assess clinical signs in some RB patients, revealing a retinal mass with disorganization at the site of the tumor, sometimes accompanied by intratumor cavities or calcifications, subretinal fluid, retinal edema or thinning, and epiretinal membrane formation (24, 25). Genetic testing has been used in retinoblastoma and other genetic diseases. Genetic testing provides insight into the inheritance of parental *RB1* mutant alleles, offering several options for further action. Knowledge of the patient’s *RB1* mutation profile enables precise screening of subsequent generations and relatives. Once the familial *RB1* mutation is known, screening can also be performed at any stage of the pregnancy (3). Recent guidelines emphasize that updated functional classification of *RB1* variants heavily informs surveillance recommendations for predisposed children (26, 27).

The risk that offspring will inherit the mutant *RB1* gene from an affected parent is 50%, which results in a 97% risk of developing RB and a high lifelong risk of developing other cancers (4). The sensitivity is approximately 95% in molecular genetic testing for *RB1* mutations (3). With the development of gene therapy, gene mutation analysis is becoming an important tool for genetic counseling and prenatal diagnosis. It is expected that detection of mutations will become more established in the future. Timely and early detection along with the education of the parents and physicians will help reduce morbidity and the occurrence of secondary tumors in RB patients, which will lead to better therapeutic effects for RB patients. The identification of the *RB1* hotspot mutation site will promote the diagnosis and therapy of retinoblastoma.

## Data Availability

The original contributions presented in the study are publicly available. This data can be found here: Zenodo, [https://doi.org/10.5281/zenodo.20149363], Accession number: [10.5281/zenodo.20149362].
